# Lobeglitazone, a novel thiazolidinedione, for secondary prevention in patients with ischemic stroke: a nationwide nested case-control study

**DOI:** 10.1186/s12933-023-01841-4

**Published:** 2023-05-05

**Authors:** Joonsang Yoo, Jimin Jeon, Minyoul Baik, Jinkwon Kim

**Affiliations:** Department of Neurology, Yongin Severance Hospital, Yonsei University College of Medicine, 363 Dongbaekjukjeon-daero, Giheung-gu, Yongin-si, Gyeonggi-do 16995 Republic of Korea

**Keywords:** Type 2 Diabetes Mellitus, Cardiovascular Disease, Cohort Study, Thiazolidinedione, Lobeglitazone

## Abstract

**Introduction:**

Ischemic stroke patients with diabetes are at high risk for recurrent stroke and cardiovascular complications. Pioglitazone, a type of thiazolidinedione, has been shown to reduce cardiovascular complications in patients with ischemic stroke and type 2 diabetes (T2D) or insulin resistance. Lobeglitazone is a novel thiazolidinedione agent that improves insulin resistance and has similar glycemic efficacy to pioglitazone. Using population-based health claims data, we evaluated whether lobeglitazone has secondary cardiovascular preventive effects in patients with ischemic stroke and T2D.

**Methods:**

This study has a nested case-control design. From nationwide health claims data in Korea, we identified patients with T2D admitted for acute ischemic stroke in 2014–2018. Cases were defined who suffered the primary outcome (a composite of recurrent stroke, myocardial infarction, and all-cause death) before December 2020. Three controls were selected by incidence density sampling for each case from those who were at risk at the time of their case occurrence with exact matching on sex, age, the presence of comorbidities, and medications. As a safety outcome, we also evaluated the risk of heart failure (HF) according to the use of lobeglitazone.

**Results:**

From the cohort of 70,897 T2D patients with acute ischemic stroke, 20,869 cases and 62,607 controls were selected. In the multivariable conditional logistic regression, treatment with lobeglitazone (adjusted OR 0.74; 95% CI 0.61–0.90; p = 0.002) and pioglitazone (adjusted OR 0.71; 95% CI 0.64–0.78; p < 0.001) were significantly associated with a lower risk for the primary outcome. In a safety outcome analysis for HF, treatment with lobeglitazone did not increase the risk of HF (adjusted OR 0.90; 95% CI 0.66–1.22; p = 0.492).

**Conclusions:**

In T2D patients with ischemic stroke, lobeglitazone reduced the risk of cardiovascular complications similar to that of pioglitazone without an increased risk of HF. There is a need for further studies on the cardioprotective role of lobeglitazone, a novel thiazolidinedione.

**Supplementary Information:**

The online version contains supplementary material available at 10.1186/s12933-023-01841-4.

## Background

Stroke is the leading cause of death and disability worldwide. Type 2 diabetes mellitus (T2D) is characterized by insulin resistance and β-cell dysfunction, which is a strong independent risk factor for stroke and is a very prevalent comorbidity in patients with stroke [1]. Stroke patients with T2D have worse prognoses and higher risks for recurrent cardiovascular events than those without diabetes [2]. Guidelines for the secondary prevention of stroke recommend proper glycemic control with multifaceted lifestyle interventions and antidiabetic agents for stroke patients with diabetes who are at high-risk for recurrent cardiovascular complications [3]. Experimental and epidemiologic data have suggested that some classes of antidiabetic medications have cardiovascular protective action beyond the glucose-lowering effect [4, 5]. Pioglitazone has been proven to reduce cardiovascular complications in patients with ischemic stroke [6–9]. It is a thiazolidinedione-type drug that acts as an insulin sensitizer through activation of the peroxisome proliferator-activated receptor-γ (PPARγ), a nuclear hormone receptor that plays a key role in regulating energy homeostasis, anti-inflammation, lipid/glucose metabolism, and adipocyte function [10].

Lobeglitazone (Chong Kun Dang Pharmaceutical Corporation, Seoul, Korea) is a newly developed thiazolidinedione [11]. Compared to pioglitazone and other thiazolidinediones, lobeglitazone has a higher affinity to PPARγ [12]. Considering the favorable safety and glucose-lowering effect of lobeglitazone in experimental and clinical trials, it has been approved as an oral antidiabetic agent and has been used in the treatment of T2D in Korea since July 2013 [13–15]. Given the established secondary preventive effect of pioglitazone, lobeglitazone, another thiazolidinedione-based PPARγ agonist, likely plays a protective role in stroke patients with T2D. To evaluate the potential secondary preventive role of lobeglitazone, we performed a population-based nested case-control study on the development of recurrent cardiovascular events in patients with acute ischemic stroke and T2D. As a safety outcome, we also investigated whether the use of lobeglitazone increases the risk of heart failure (HF).

## Methods

### Study design and data source

We conducted a nested case-control study based on the nationwide health claims data in Korea. Korea has a national single-payer healthcare system, the National Health Insurance System (NHIS), which covers almost the entire population in Korea [16]. The Health Insurance Review and Assessment Service (HIRA) is an independent agency for claims review and quality assessment of the national health insurance service. All paper- and electronic-based health claims submitted by service providers are reviewed by the HIRA and sent to the NHIS. The HIRA has opened a nationwide health claims database to researchers for academic and political purposes [17]. The HIRA dataset contains the hospital visits, medical procedures, prescription records, diagnostic codes, demographics, and death statistics of the study population [18]. Diagnostic codes are recorded based on the International Classification of Diseases, 10th Revision (ICD-10). The HIRA dataset was fully anonymized to protect personal information and privacy, and access to the dataset is only available through the cloud system, so the researchers did not extract any personal data. There is an increasing number of publications with clinical research using the Korean nationwide healthcare claims data. Due to the nature of a retrospective study based on fully anonymized data, this study was approved, and the requirement for informed consent was waived by the Institutional Review Board of Yongin Severance Hospital (9-2021-0096).

### Study participants

**Fig. 1:**
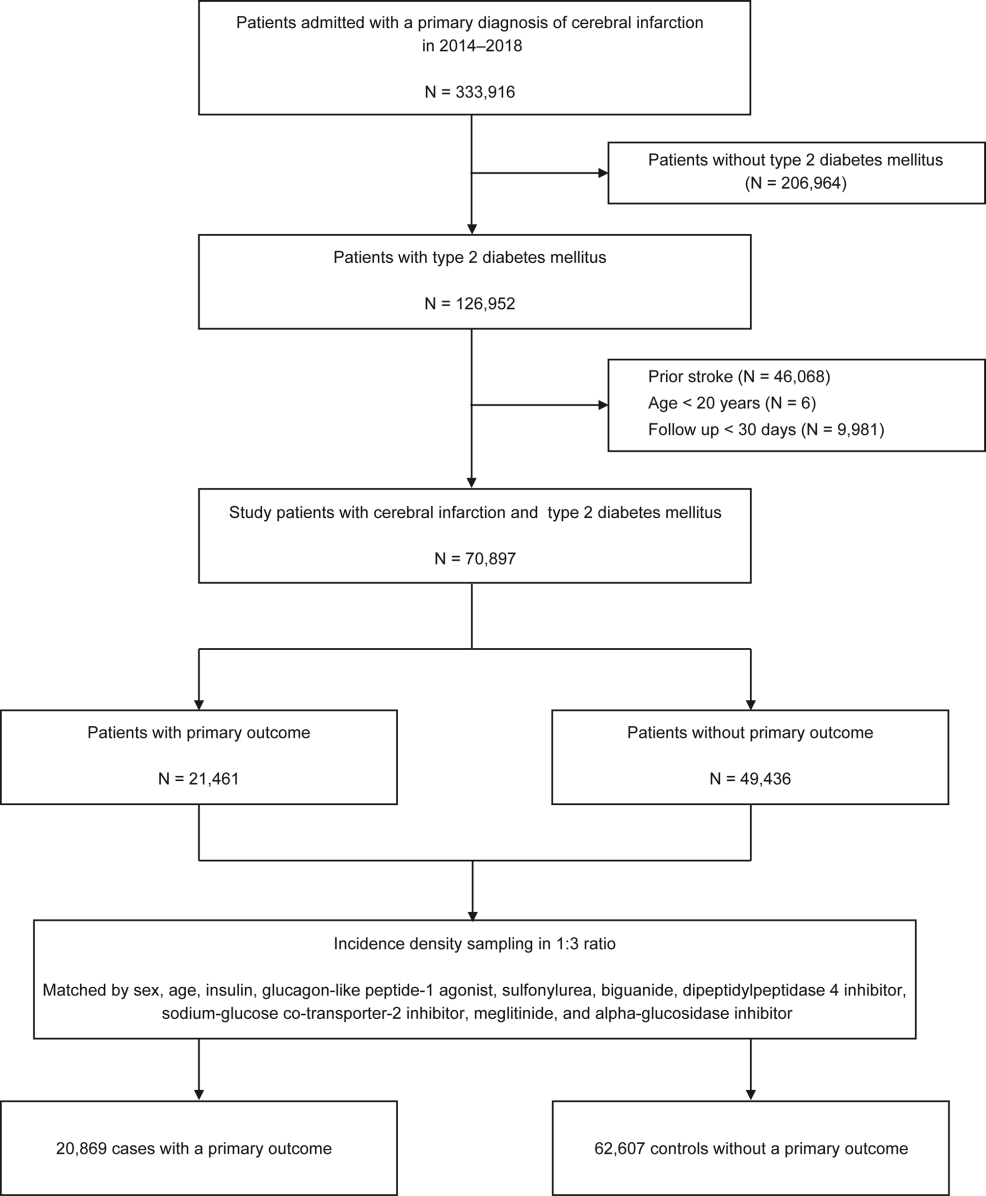
Flow chart for the selection of cases and controls in the nested case-control study for primary outcome

From the nationwide health claims data from HIRA, we constructed a cohort of patients aged ≥ 20 years old with T2D who were admitted with the primary diagnosis of acute ischemic stroke (ICD-10 code of I63) and completed brain CT or MRI during hospital admission between 2014 and 2018. In the cohort, the index date of each patient was the admission date of the index stroke. The presence of T2D was determined by the patient having at least one claim for antidiabetic medications with the related diagnostic code (E11, E12, E13, or E14) before the discharge of index stroke [19]. Based on the health claims data, the study patients were followed up until the development of a primary outcome, death, loss of eligibility for health insurance, or the study end date (December 31, 2020). To only include patients with acute ischemic stroke, we excluded those who had prior diagnostic codes of stroke (I60–64 or I69) before the index stroke. Patients with too short of a follow-up period (< 30 days) were also excluded (Fig. 1).

### Primary outcome

The primary outcome is a composite of the development of recurrent stroke, MI, and all-cause death after index stroke. In order to exclude complications of the index stroke itself, only events that occurred 30 days after the index stroke were collected. Using the HIRA dataset, the development of the primary outcome was evaluated until Dec 31, 2020. The development of recurrent stroke was defined as admission with a primary diagnosis of I60–63 accompanied by brain CT or MRI [20]. The development of MI was defined as admission with a primary diagnosis of I21 [21]. In the NHIS, the diagnostic accuracy of I60–I63 for stroke and I21 for MI has been validated in prior studies [22, 23]. The date of death was inquired from death records in the HIRA database. If patients had multiple outcomes, only the earliest outcome was considered. If death and stroke (or MI) occurred on the same day, the primary outcome was considered stroke (or MI). In a secondary outcome analysis for the individual outcomes, cases were selected only from those who experienced the outcome of interest first.

### Selection of cases and controls

To construct a nested case-control study dataset, we defined cases as patients who suffered from the primary outcome (recurrent stroke, MI, and all-cause death) during the study follow-up period. We selected patients with a primary outcome as the case group and used incidence density sampling to select three controls from the cohort. Controls were selected with replacement from the dynamic risk set at the time of case occurrence, where all patients who were event-free and at risk were eligible, except for the case itself (Supplemental Fig. 1). Controls were also fully matched on the same sex and age (± 1 year allowed) and were taking the same antidiabetic medications (insulin, glucagon-like peptide-1 agonist, sulfonylurea, biguanide, dipeptidyl peptidase 4 inhibitor, sodium-glucose co-transporter-2 inhibitor, meglitinide, alpha-glucosidase inhibitor), except for thiazolidinediones (pioglitazone or lobeglitazone), at the time of their matched case.

### Covariates

We collected data on sex, age, and the presence of risk factors at the index stroke. The presence of hypertension, atrial fibrillation, coronary artery disease, renal disease, and malignancy was evaluated by the presence of the related diagnostic and claims codes before or at the index stroke in the HIRA dataset. Hypertension was considered if the patients had the corresponding ICD-10 codes (I10–13 or I15) with a prescription of antihypertensive drugs. Atrial fibrillation was determined by the presence of ICD-10 code I48. Coronary artery disease was defined as the presence of ICD-10 codes (I20–25) as a main diagnosis or claim codes for percutaneous coronary intervention (M6551–2, M6561–4, and M6571–2) or coronary artery bypass graft (O1641–2, O1647, OA641–2, and OA647) [24]. Renal disease was determined by the presence of the related diagnostic codes (ICD-10 codes N17–19, E08.2, E10.2, E11.2, E13.2 or I12–13) or claims of hemodialysis or peritoneal dialysis [20]. Malignancy was identified if patients had the corresponding ICD-10 code of C00–C97 with a special code (V027, V193, and V194) applied for economic benefits available to confirmed cancer patients in the HIRA [25].

### Assessment of medications

Medication usage during the longitudinal period typically varies with time, and treatment with antidiabetic medications frequently changes in practice. Therefore, we investigated the use of antidiabetic medications and common cardiovascular medications as covariates at the time the primary outcome occurred in the case group or the matched time in the control group. In Korea, these medications should be prescribed by a physician, and the prescription records (prescription date, drug name, dosage, and duration) are available in the HIRA claims database. Treatment with thiazolidinediones (lobeglitazone, pioglitazone) and other classes of oral antidiabetic medications (sulfonylurea, biguanide, dipeptidyl peptidase 4 inhibitor, sodium-glucose co-transporter-2 inhibitor, meglitinide, and alpha-glucosidase inhibitor) was determined whether exposure to the medications within 7 days from the time of primary outcome or the matched time. Because the duration of parenteral antidiabetic medications (insulin and glucagon-like peptide-1 agonist) could not be directly evaluated from the prescription record (example of insulin prescription: [regular insulin, 100 units/mL, 10 mL Vial] × 2 on May 11, 2019) unlike oral medications, treatment with insulin and glucagon-like peptide-1 agonist was determined by the presence of the prescriptions within the past 90 days [9]. As covariates, we also evaluated exposure to oral antiplatelets (aspirin, clopidogrel, ticlopidine, prasugrel, ticagrelor, triflusal, and cilostazol), oral anticoagulants (coumadin, rivaroxaban, apixaban, edoxaban, and dabigatran), and statins (atorvastatin, fluvastatin, lovastatin, pitavastatin, pravastatin, rosuvastatin, and simvastatin) within 7 days of the primary outcome or the matched time.

### Safety outcome

HF is the most worrisome adverse effect of thiazolidinediones [26]. As a safety outcome, we evaluated the risk of HF in the study patients without a prior diagnosis of HF. The development of HF was defined as when the patients had a health insurance claim record with a primary or secondary diagnosis of HF (I11.0, I13.0, I13.2, and I50.x) during the follow-up period [27]. Of them, we constructed an additional nested-case control study dataset for the development of HF. As was done for the primary analysis, for cases of HF, we selected three controls from those matched by sex and age (± 1 year allowed) and antidiabetic medications, except thiazolidinediones.

### Statistical analysis

Categorical variables are summarized as number (proportion) and continuous variables are expressed as mean ± standard deviation (SD). We constructed a conditional logistic regression model with case-control groups matched for sex, age, and concomitant antidiabetic medications except for thiazolidinediones. Compared to ‘no thiazolidinedione’, we calculated the odds ratio (OR) and 95% confidence interval (CI) for ‘lobeglitazone’ and ‘pioglitazone’. Adjustments were made for covariates (hypertension; atrial fibrillation; renal disease; coronary artery disease; malignancy; and the use of oral antiplatelets, oral anticoagulants, and statin). Data manipulation and statistical analyses were performed with SAS statistical software (version 9.4.2; SAS Institute Inc., Cary, NC, USA) and R software (version 3.5.1; R Foundation for Statistical Computing, Vienna, Austria; http://www.r-project.org/). A two-sided p value of < 0.05 was considered statistically significant.

## Results

### The study cohort of patients with acute ischemic stroke and T2D

From the HIRA database, we found 126,952 patients with T2D who were admitted with a primary diagnosis of ischemic stroke from 2014 to 2018. In accordance with the inclusion and exclusion criteria, we constructed a cohort of 70,897 T2D patients with acute ischemic stroke. During the 3.31 ± 1.91 years (mean ± SD) of the post-stroke follow-up period, there were 21,461 patients (30.3%) who suffered the primary outcome (recurrent stroke, MI, or all-cause death). With the use of a nested case-control approach and 1:3 incidence density sampling, we finally selected 20,869 cases with the primary outcome and 62,607 matched controls without the primary outcome. Figure 1 shows a schematic diagram outlining the process of case and control selection for a nested case-control study.

### Results of the nested case-control study

The characteristics of the selected 20,869 cases and 62,607 controls are shown in Table 1. Due to the exact matching process between the cases and the controls, there were no differences in sex, age, and treatment with antidiabetic medications except for thiazolidinediones. In the control group, treatment with thiazolidinediones (pioglitazone or lobeglitazone) was more frequent than cases (4.4% vs. 3.1%), suggesting a lower risk of primary outcome with thiazolidinediones. In the multivariable conditional logistic regression adjusted for covariates (Table 2), treatment with lobeglitazone (adjusted OR 0.74; 95% CI 0.61–0.90; p = 0.002) and treatment with pioglitazone (adjusted OR 0.71; 95% CI 0.64–0.78; p < 0.001) were associated with a lower risk of primary outcome occurrence compared to those not administered thiazolidinediones. When pioglitazone was set as the reference, the risk of primary outcome did not significantly differ between those treated with lobeglitazone and pioglitazone (adjusted OR 1.05; 95% CI 0.85–1.30 for lobeglitazone compared to pioglitazone; p = 0.605).

**Table 1:** Characteristics of the cases and matched controls for primary outcome

Variable	Case (n = 20,869)	Control (n = 62,607)
Duration between index stroke and the development of the case, year	1.70 ± 1.54	1.70 ± 1.54
Sex, male	11,710 (56.11)	3,5130 (56.11)
Age at index stroke, years	72.21 ± 10.84	72.18 ± 10.81
Comorbidities		
Hypertension	18,971 (90.91)	54,801 (87.53)
Atrial fibrillation	5,507 (26.39)	12,227 (19.53)
Malignancy	3,158 (15.13)	5,619 (8.98)
Renal disease	7,436 (35.63)	18,854 (30.12)
Coronary artery disease	4,137 (19.82)	10,882 (17.38)
Non-oral antidiabetic medication		
Insulin	5,983 (28.67)	17,949 (28.67)
Glucagon-like peptide-1 agonist	3 (0.01)	9 (0.01)
Oral antidiabetic medication		
Sulfonylurea	4,038 (19.35)	12,114 (19.35)
Biguanide	7,702 (36.91)	23,106 (36.91)
Dipeptidyl peptidase 4 inhibitor	6,352 (30.44)	19,056 (30.44)
Sodium glucose co-transporter-2 inhibitor	222 (1.06)	666 (1.06)
Meglitinide	18 (0.09)	54 (0.09)
Alpha-glucosidase inhibitor	148 (0.71)	444 (0.71)
Cardiovascular medication		
Antiplatelet	11,119 (53.28)	40,125 (64.09)
Anticoagulant	2,306 (11.05)	6,876 (10.98)
Statin	9,901 (47.44)	37,640 (60.12)
Thiazolidinedione treatment		
no thiazolidinedione	20,226 (96.92)	59,823 (95.55)
Lobeglitazone	135 (0.65)	559 (0.89)
Pioglitazone	508 (2.43)	2,225 (3.55)

Data are shown as number (%) and mean ± standard deviation

Cases and controls (1:3) are matched for sex, age (allowed for ± 1 year), and treatment with insulin, glucagon-like peptide-1 agonist, sulfonylurea, biguanide, dipeptidyl peptidase 4 inhibitor, sodium-glucose co-transporter-2 inhibitor, meglitinide, and alpha-glucosidase inhibitor

**Table 2:** Risk factors for the primary outcome in patients with acute ischemic stroke and type 2 diabetes mellitus

	Adjusted OR [95% CI]	P value
Comorbidities		
Hypertension	1.35 [1.28–1.43]	< 0.001
Atrial fibrillation	1.63 [1.56–1.71]	< 0.001
Malignancy	1.84 [1.75–1.93]	< 0.001
Renal disease	1.26 [1.21–1.30]	< 0.001
Coronary artery disease	1.19 [1.14–1.24]	< 0.001
Cardiovascular medication		
Antiplatelet	0.73 [0.70–0.76]	< 0.001
Anticoagulant	0.62 [0.58–0.66]	< 0.001
Statin	0.63 [0.60–0.65]	< 0.001
Thiazolidinedione treatment		
no thiazolidinedione	ref	
Lobeglitazone	0.74 [0.61–0.90]	0.002
Pioglitazone	0.71 [0.64–0.78]	< 0.001

OR; odds ratio, CI; confidence interval

The primary outcome is defined as a composite of recurrent stroke, myocardial infarction, and all‑cause death after acute ischemic stroke

Data are derived from multivariable conditional logistic regression analysis adjusted for the listed comorbidities, the use of cardiovascular medications, and thiazolidinediones with the matched case-control dataset described in Table 1

### Secondary outcome analysis

Among the 20,869 cases with the primary outcome, the number of patients with recurrent stroke, MI, and all-cause death after index stroke was 10,060, 951, and 9,858, respectively. To evaluate the effect of lobeglitazone on the secondary outcomes, we constructed three nested case-control groups consisting of cases with individual outcomes and matched controls (Table 3). In the secondary outcome analysis, treatment with lobeglitazone was associated with a reduced risk of recurrent stroke, MI, and all-cause death, but statistical significance was only found for all-cause death.

**Table 3:** Individual outcome analysis according to treatment with pioglitazone and lobeglitazone

Outcome (number of case patients with the outcome)	Primary outcome (N = 20,869)	Stroke (N = 10,060)	Myocardial infarction (N = 951)	All-cause mortality (N = 9,858)
Thiazolidinedione treatment				
no thiazolidinedione	ref	ref	ref	ref
Lobeglitazone	0.74 [0.61–0.90], p = 0.002	0.85 [0.68–1.08], p = 0.186	0.70 [0.33–1.47], p = 0.344	0.58 [0.39–0.86], p = 0.006
Pioglitazone	0.71 [0.64–0.78], p < 0.001	0.78 [0.69–0.89], p < 0.001	0.71 [0.47–1.08], p = 0.111	0.60 [0.49–0.72], p < 0.001

Data are adjusted odds ratios and [95% confidence intervals] obtained from multivariable conditional logistic regression analyses adjusted for the covariables described in Table 2 with the matched case-control dataset described in Table 1

### Sensitivity analysis considering the burden of antidiabetic medications

In the nested case-control design matched for antidiabetic agents except for thiazolidinediones, there is a potential concern that the beneficial effect of pioglitazone may reflect the treatment with multiple antidiabetic agents being more aggressive rather than the class effect of thiazolidinediones (the difference in risk between biguanide and biguanide plus lobeglitazone may be due to the higher medication burden rather than lobeglitazone itself). As a sensitivity analysis to evaluate this concern, we reconstructed another nested case-control model (Supplementary Fig. 2) in which each case was matched with 3 controls for sex, age, the use of parenteral antidiabetic agents, and the number of concurrent oral antidiabetic agents (sulfonylurea, biguanide, dipeptidyl peptidase 4 inhibitor, sodium-glucose co-transporter-2 inhibitor, meglitinide, alpha-glucosidase inhibitor, and thiazolidinedione). For example, a patient taking biguanide plus lobeglitazone could be matched with a patient taking biguanide plus dipeptidyl peptidase 4 inhibitor. In the model matched for the number of oral antidiabetic medications taken (Supplementary Table 1), the reduced risk for primary outcome development with lobeglitazone (adjusted OR 0.79; 95% CI 0.65–0.97; p = 0.021) and pioglitazone (adjusted OR 0.76; 95% CI 0.69–0.85; p < 0.001) was consistent.

### Safety outcome analysis for HF

For a safety outcome analysis, we created a nested case-control dataset for HF from a cohort of patients without prior HF diagnoses (Supplementary Fig. 3). Among the 60,859 patients without prior HF, there were 5,111 patients who developed HF during the study follow-up period. Using a multivariable conditional logistic regression analysis with the 1:3 matched case-control dataset, treatment with lobeglitazone did not have a significant association with the risk of HF (Supplementary Table 2). Compared to no thiazolidinedione, the adjusted OR [95% CI], p-value for lobeglitazone and pioglitazone were 0.90 [0.66–1.22], p = 0.492 and 1.15 [0.98–1.35], p = 0.079, respectively.

## Discussion

In this population-based nested case-control study, we found that treatment with lobeglitazone was associated with a lower risk of secondary cardiovascular complications in T2D patients with acute ischemic stroke. The secondary preventive effect of lobeglitazone was similar to that of pioglitazone, a thiazolidinedione well-established to be effective in reducing recurrent stroke and major vascular events in ischemic stroke patients [28]. The beneficial effect of lobeglitazone remained significant in the control of other antidiabetic medications and traditional cardiovascular medications, such as antithrombotics and statins. In a safety analysis, treatment with lobeglitazone did not increase the risk of HF.

Patients who survived ischemic stroke are at high risk of recurrent stroke or cardiovascular complications. One in four stroke survivors experience a second stroke within five years, and as many as 30% of all strokes are secondary strokes [29]. The risk of recurrence is further increased in patients with cardiovascular risk factors, such as T2D or insulin resistance [30]. Therefore, it is crucial to establish effective cardiovascular prevention strategies for the high-risk groups of ischemic stroke patients with T2D [3]. Besides lowering the level of hemoglobin A1c, recent guidelines for T2D recommend that the selection and use of antidiabetic agents should address cardiovascular risk as well as glycemic control [28, 31].

In the case of stroke patients, the cardiovascular protective role of pioglitazone has been well-established for more than a decade [8, 28]. Pioglitazone is the most widely used thiazolidinedione and acts as an insulin sensitizer through the activation of PPARγ. The PROspective pioglitAzone Clinical Trial In macroVascular Events (PROactive) trial showed that pioglitazone reduced the risk of fatal or nonfatal stroke (hazard ratio 0.53; 95% CI 0.34–0.85) in T2D patients with prior stroke [8]. In the Insulin Resistance Intervention After Stroke Trial (IRIS), those taking pioglitazone with good adherence had reduced their risk for stroke by 33%, and acute coronary syndrome by 52% over a median follow-up of 4.8 years [32]. In a meta-analysis of stroke patients with three randomized controlled trials, treatment with pioglitazone was significantly associated with a reduced risk of recurrent stroke (hazard ratio 0.68; 95% CI 0.50–0.92) and major vascular events (hazard ratio 0.75; 95% CI 0.64–0.87) [33]. There are numerous examples of experimental and clinical evidence for the vascular protective role of pioglitazone in reducing atherosclerosis progression, atherosclerotic plaque inflammation, in-stent restenosis after coronary artery stent implantation, progression rate from persistent to permanent atrial fibrillation, and repeated ablation rate in patients with paroxysmal atrial fibrillation after catheter ablation [34–36]. These data suggest that pioglitazone, a thiazolidinedione that is an insulin sensitizer by activating PPARγ, should be used more widely for cardiovascular prevention in high-risk patients, especially those with a history of stroke [32, 37].

Currently, lobeglitazone and pioglitazone are two available thiazolidinediones for T2D in Korea [11]. Like other thiazolidinediones, lobeglitazone promotes adipocyte differentiation, increases glucose uptake, and decreases pro-inflammatory responses, which leads to improved insulin sensitivity by PPARγ activation [38, 39]. The activation of PPARγ promotes fatty acid uptake, triglyceride formation, and storage in lipid droplets, thereby increasing insulin sensitivity and glucose metabolism [40]. PPARγ also exerts anti-inflammatory, anti-proliferative, and antiatherogenic effects on the vascular wall and immune cells, which can reduce cardiovascular risk. Considering the accumulating evidence, PPARγ has emerged as one of the promising therapeutic targets for cardiovascular disease [41]. Lobeglitazone displays 12 times higher affinity to PPARγ than other thiazolidinediones [12, 42]. Owing to its higher affinity to PPARγ, lobeglitazone (0.5 mg/day) has similar efficacy regarding glycemic control with a 30-times smaller dose compared to pioglitazone (15 mg/day) [11, 43]. In a real-world observational study, treatment with 0.5 mg lobeglitazone had a good long-term safety profile with an apparent reduction in glycosylated hemoglobin; decreased levels of total cholesterol, triglyceride, and low-density lipoprotein cholesterol; and increased high-density lipoprotein cholesterol, suggesting both glucose-lowering and lipid-modifying effects [44]. Lobeglitazone treatment for T2D patients with nonalcoholic fatty liver disease reduced intrahepatic fat content and improved glycemic, liver, and lipid profiles [45]. Lobeglitazone is also effective in reducing albuminuria, a well-known marker of the increased risk of renal and cardiovascular disease [46]. In experimental studies, lobeglitazone exerted anti-inflammatory and anti-atherosclerotic potentials like pioglitazone [38, 47]. Our current data added clinical evidence that lobeglitazone, a novel thiazolidinedione, could be a good treatment option for T2D patients at high cardiovascular risk. There is a need for further studies on the pathophysiologic and practical role of lobeglitazone while controlling for residual cardiovascular risk [48].

Even with the promising and proven cardiovascular preventive effects of pioglitazone, the use of pioglitazone for stroke treatment is not frequent in clinical practice [48–50]. Indeed, the proportion of patients taking pioglitazone is low (< 4%) in our nationwide data. The main cause of the low use of pioglitazone in clinical practice is the concern for potential side effects, particularly the risk of HF [36, 49, 50]. In the current study, we did not find any evidence of an increased risk of HF with lobeglitazone in patients without a prior diagnosis of HF. Additional research is needed, but our data suggest that lobeglitazone could be a good choice of thiazolidinediones not frequently used in practice due to the concern of HF despite the established cardiovascular benefits.

### Advantages and limitations

The current study has several advantages and limitations. Using the nationwide health claims data, we could collect a large number of ischemic stroke patients with T2D in real-world practice. Evaluating a nationwide claims database, we were able to investigate the long-term incidence of cardiovascular complications of them. Because Korea has a public, single-payer health insurance system, and antidiabetic medications should be prescribed by a physician, all prescription data is available in the HIRA database. Based on the prescription data, we could get detailed information about the medications during a long-term follow-up period in individual patients. Ischemic stroke patients with T2D frequently take a combination of antithrombotics, statins, and multiple classes of antidiabetic medications. To reduce the potential bias with concomitant medications, we conducted a nested case-control study design that matched for individual antidiabetic medications and adjusted for the use of antithrombotics and statins. Our study findings showed a reduced risk for the primary outcome with the use of antithrombotics and statins and an increased risk of the primary outcome with concurrent risk factors. These findings were consistent with prior epidemiologic knowledge, which suggests the reliability of our data. As well as cardiovascular outcome, we evaluated the risk of HF, the most potential side effect and the reason for the reluctance to use the thiazolidinedione class.

We should also address the limitations of a retrospective study design based on a pre-existing health claims database. The information in the claims database is not made for clinical research purposes. There is lacked clinical information for important traditional risk factors such as smoking, physical activity, blood pressure, laboratory results such as lipid profiles or inflammatory markers, the severity of index stroke, duration of T2D, body weight, and the level of hemoglobin A1c, a marker of good glycemic control. This study was performed with only Korean patients with ischemic stroke and T2D. There might be racial and ethnic disparities in the characteristics of the stroke patients, health care delivery system, or the response to medications [51]. We should also consider the possibility of hidden bias between patients who received lobeglitazone and those who did not. In addition, there might be a gap between prescription data and the actual intake of medications. Diagnosis of stroke/MI based on the Korean health claims data is known to be accurate, but since the events were defined as only hospitalized cases, those with cardiovascular complications who did not admit to the hospital may not have been captured. As the development of HF was determined based on the diagnostic code, we only evaluated the risk of HF in patients without a prior diagnosis of HF. We could not access clinical information such as echocardiography or patient symptoms related to HF. While our study did not find an association between lobeglitazone and the risk of HF, there was a possibility of selection bias that lobeglitazone was less frequently prescribed to individuals considered a high risk of HF. Thus, further research is needed to investigate the potential role of lobeglitazone in the development of HF.

## Conclusions

In T2D patients with ischemic stroke, treatment with lobeglitazone was associated with a reduced risk of cardiovascular complications including recurrent stroke, MI, and all-cause mortality similar to that of pioglitazone. There was no increased risk of HF with the use of lobeglitazone. Further research is needed on the cardioprotective role of lobeglitazone, a novel thiazolidinedione.

## Electronic supplementary material

Below is the link to the electronic supplementary material.

Supplementary Material 1

## Data Availability

The dataset supporting the results of this study is accessible from HIRA in Korea, but with restrictions to data availability. The use of the dataset is restricted to the current research under license; therefore, public access of the dataset is not available. Researchers are only access the data upon reasonable request with approval from the inquiry committee of research support in HIRA (https://opendata.hira.or.kr/or/orb/useGdInfo.do).

## List of Abbreviations

CI: Confidence interval
CVD: Cardiovascular disease
HIRA: Health Insurance Review and Assessment Service
HR: Hazard ratio
ICD-10: The 10th edition of the International Statistical Classification of Diseases
MI: Myocardial infarction
OR: Odds ratio
PPARγ: Peroxisome proliferator-activated receptor-γ
T2D: Type 2 diabetes mellitus
