# Supplementary material for: Lobeglitazone, a novel thiazolidinedione, for secondary prevention in patients with ischemic stroke: a nationwide nested case-control study
Source: Cardiovasc Diabetol. 2023 May 5;22:106. doi: 10.1186/s12933-023-01841-4 (PMC10163714; doi:10.1186/s12933-023-01841-4)
Supplement: Supplementary file 1 — Supplementary Material 1 [file 12933_2023_1841_MOESM1_ESM.docx]

**SUPPLEMENTARY MATERIALS**

**Title**: Lobeglitazone, a novel thiazolidinedione, for secondary prevention in patients with ischemic stroke: a nationwide nested case-control study

Running title: Lobeglitazone for secondary prevention after ischemic stroke

Joonsang Yoo^1^, Jimin Jeon^1^, Minyoul Baik^1^, Jinkwon Kim^1^

^1^Department of Neurology, Yongin Severance Hospital, Yonsei University College of Medicine, 363 Dongbaekjukjeon-daero, Giheung-gu, Yongin-si, Gyeonggi-do16995, Republic of Korea

**Corresponding author:**

Jinkwon Kim, MD, PhD

Department of Neurology, Yongin Severance Hospital, Yonsei University College of Medicine, 363 Dongbaekjukjeon-daero, Giheung-gu, Yongin-si, Gyeonggi-do 16995, Republic of Korea

Tel: +82-31-5189-8152; Fax: +82-31-5189-8208; E-mail: [antithrombus@yuhs.ac](mailto:antithrombus@yuhs.ac); [antithrombus@gmail.com](mailto:antithrombus@gmail.com)

**Supplemental Figure 1.** Schematic design of 1:3 nested case-control design using incidental density sampling.


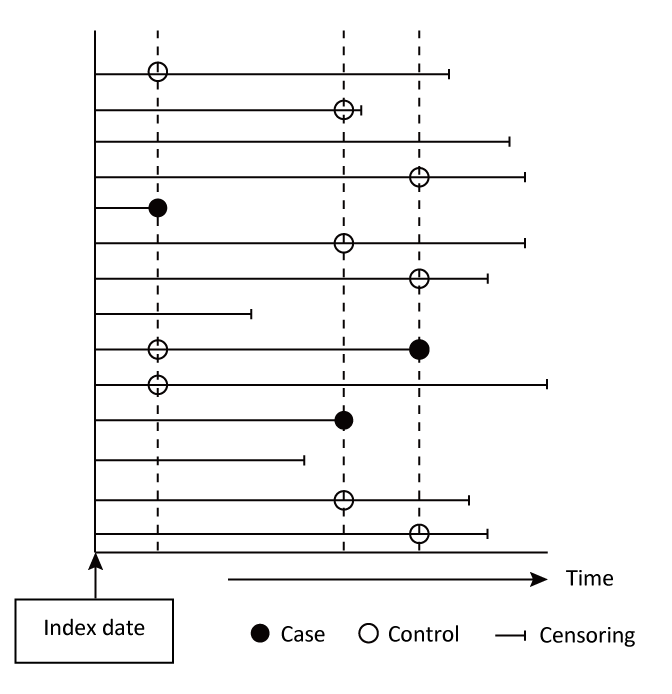


**Supplementary Figure 2.** Flow chart for the selection of cases and controls in the nested case-control study for primary outcome matched by number of concurrent oral antidiabetic agents


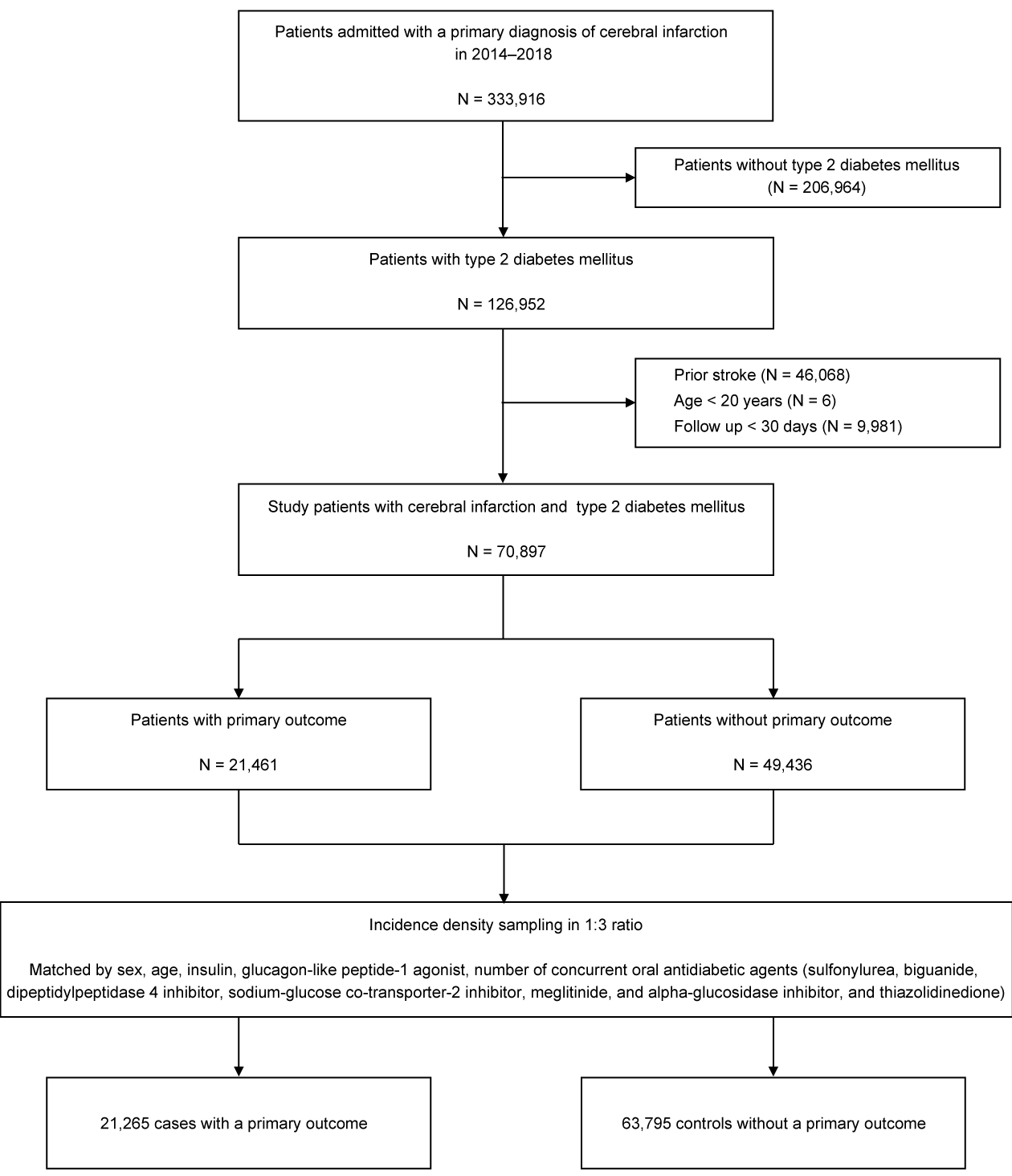


**Supplementary Figure 3.** Flow chart for the selection of cases and controls in the nested case-control study for heart failure

**
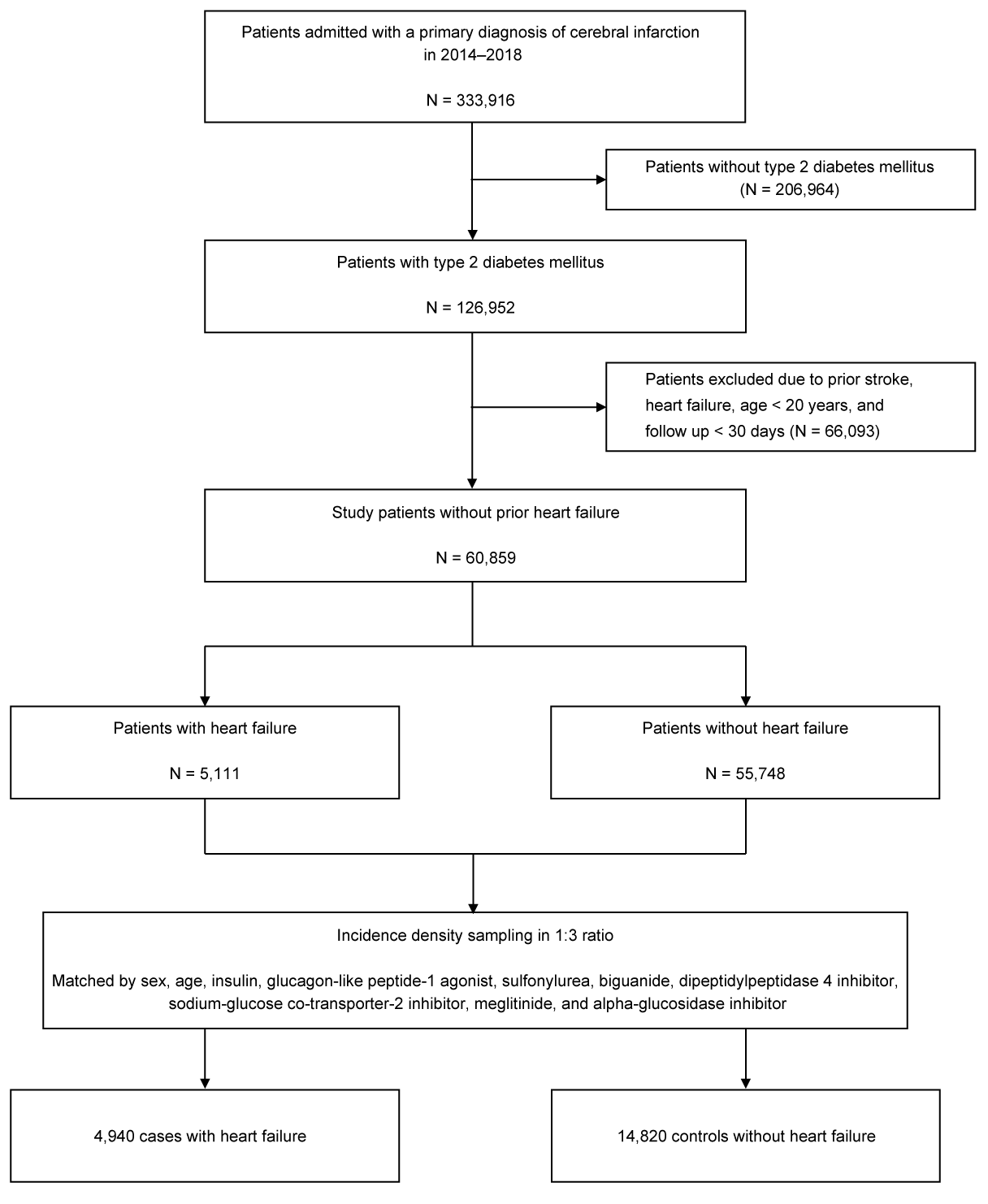
**

**Supplementary Table 1.** Characteristics of the cases and matched controls for primary outcome regarding the number of concurrent oral antidiabetic agents

| Variable | Case (n=21,265) | Control (n=63,795) | Crude OR [95% CI] | Adjusted OR [95% CI] | P value^*^ |
| --- | --- | --- | --- | --- | --- |
| Duration between index stroke and development of case, year | 1.71±1.56 | 1.71±1.56 | - | - |  |
| Sex, male | 11926 (56.08) | 35778 (56.08) | - | - |  |
| Age, years | 72.19±10.93 | 72.17±0.90 | - | - |  |
| Comorbidities |  |  |  |  |  |
| hypertension | 19332 (90.91) | 56117 (87.97) | 1.38 [1.31–1.46] | 1.30 [1.23–1.37] | <0.001 |
| atrial fibrillation | 5593 (26.30) | 12458 (19.53) | 1.49 [1.44–1.55] | 1.64 [1.56–1.72] | <0.001 |
| malignancy | 3224 (15.16) | 5603 (8.78) | 1.89 [1.80–1.98] | 1.88 [1.79–1.97] | <0.001 |
| renal disease | 7621 (35.84) | 18964 (29.73) | 1.34 [1.30–1.39] | 1.30 [1.25–1.34] | <0.001 |
| coronary artery disease | 4238 (19.93) | 11320 (17.74) | 1.16 [1.11–1.20] | 1.17 [1.12–1.22] | <0.001 |
| Non-oral antidiabetic medication |  |  |  |  |  |
| insulin | 6218 (29.24) | 18654 (29.24) | - | - |  |
| glucagon-like peptide-1 agonist | 3 (0.01) | 9 (0.01) | - | - |  |
| Number of concurrent oral antidiabetic agents |  |  |  |  |  |
| 0 | 10843 (50.99) | 32529 (50.99) | - | - |  |
| 1 | 3563 (16.76) | 10689 (16.76) | - | - |  |
| 2 | 4352 (20.47) | 13056 (20.47) | - | - |  |
| 3 | 2424 (11.40) | 7272 (11.40) | - | - |  |
| ≥4 | 83 (0.39) | 249 (0.39) | - | - |  |
| Cardiovascular medication |  |  |  |  |  |
| antiplatelet | 11402 (53.62) | 41196 (64.58) | 0.58 [0.56–0.60] | 0.72 [0.69–0.76] | <0.001 |
| anticoagulant | 2356 (11.08) | 7040 (11.04) | 1.00 [0.96–1.06] | 0.62 [0.58–0.66] | <0.001 |
| statin | 10156 (47.76) | 38646 (60.58) | 0.54 [0.52–0.56] | 0.62 [0.60–0.65] | <0.001 |
| Thiazolidinedione treatment |  |  |  |  |  |
| no thiazolidinedione | 20625 (96.99) | 61383 (96.22) | ref | ref |  |
| lobeglitazone | 137 (0.64) | 507 (0.80) | 0.78 [0.64–0.94] | 0.79 [0.65–0.97] | 0.021 |
| pioglitazone | 503 (2.37) | 1905 (2.99) | 0.76 [0.68–0.84] | 0.76 [0.69–0.85] | <0.001 |

Data are shown as number (%), mean±standard deviation, and OR (odds ratio) [95% CI (confidence interval)] for the primary outcome (a composite of recurrent stroke, myocardial infarction, and all-cause death).

Cases and controls (1:3) are matched for sex, age (allowed for ± 1 year), treatment with insulin, glucagon-like peptide-1 agonist, and the number of concurrent oral antidiabetic agents (sulfonylurea, biguanide, dipeptidyl peptidase 4 inhibitor, sodium-glucose co-transporter-2 inhibitor, meglitinide, alpha-glucosidase inhibitor, and thiazolidinedione).

OR and 95% CI are derived from conditional logistic regression analyses for the primary outcome.

^*^P-value in the multivariable model.

**Supplementary Table 2.** Characteristics of the cases and matched controls for heart failure

| Variable | Case  (n=4,940) | Control  (n=14,820) | Crude OR [95% CI] | Adjusted OR [95% CI] | P value^*^ |
| --- | --- | --- | --- | --- | --- |
| Duration between index stroke and development of case, year | 2.05±1.57 | 2.05±1.57 |  |  |  |
| Sex, male | 2723 (55.12) | 8169 (55.12) | - | - |  |
| Age, years | 69.86±10.86 | 69.86±10.83 | - | - |  |
| Comorbidities |  |  |  |  |  |
| hypertension | 4663 (94.39) | 12730 (85.90) | 2.81 [2.47–3.21] | 2.28 [2.00–2.61] | <0.001 |
| atrial fibrillation | 1435 (29.05) | 1923 (12.98) | 2.78 [2.57–3.01] | 2.25 [2.02–2.52] | <0.001 |
| malignancy | 408 (8.26) | 1156 (7.80) | 1.07 [0.95–1.20] | 1.05 [0.92–1.19] | 0.475 |
| renal disease | 1810 (36.64) | 3906 (26.36) | 1.71 [1.59–1.84] | 1.57 [1.45–1.70] | <0.001 |
| coronary artery disease | 1330 (26.92) | 1893 (12.77) | 2.51 [2.32–2.72] | 2.27 [2.09–2.47] | <0.001 |
| Non-oral antidiabetic medication |  |  |  |  |  |
| insulin | 1075 (21.76) | 3225 (21.76) | - | - |  |
| glucagon-like peptide-1 agonist | 2 (0.04) | 6 (0.04) | - | - |  |
| Oral antidiabetic medication |  |  |  |  |  |
| sulfonylurea | 1308 (26.48) | 3924 (26.48) | - | - |  |
| biguanide | 2385 (48.28) | 7155 (48.28) | - | - |  |
| dipeptidyl peptidase 4 inhibitor | 2103 (42.57) | 6309 (42.57) | - | - |  |
| sodium glucose co-transporter-2 inhibitor | 67 (1.36) | 201 (1.36) | - | - |  |
| meglitinide | 3 (0.06) | 9 (0.06) | - | - |  |
| alpha-glucosidase inhibitor | 30 (0.61) | 90 (0.61) | - | - |  |
| Cardiovascular medication |  |  |  |  |  |
| antiplatelet | 3230 (65.39) | 10423 (70.33) | 0.76 [0.71–0.82] | 1.10 [1.00–1.21] | 0.050 |
| anticoagulant | 879 (17.79) | 1106 (7.46) | 2.75 [2.49–3.03] | 1.43 [1.24–1.66] | <0.001 |
| statin | 3230 (65.39) | 9675 (65.28) | 1.01 [0.93–1.08] | 0.87 [0.80–0.95] | 0.002 |
| Thiazolidinedione treatment |  |  |  |  |  |
| no thiazolidinedione | 4618 (93.48) | 13890 (93.73) | ref | ref |  |
| lobeglitazone | 62 (1.26) | 215 (1.45) | 0.87 [0.65–1.16] | 0.90 [0.66–1.22] | 0.492 |
| pioglitazone | 260 (5.26) | 715 (4.83) | 1.10 [0.94–1.28] | 1.15 [0.98–1.35] | 0.079 |

Data are shown as number (%), mean±standard deviation, and OR (odds ratio) [95% CI (confidence interval)] for heart failure.

Cases and controls (1:3) are matched for sex, age (allowed for ± 1 year), and treatment with insulin, glucagon-like peptide-1 agonist, sulfonylurea, biguanide, dipeptidyl peptidase 4 inhibitor, sodium-glucose co-transporter-2 inhibitor, meglitinide, and alpha-glucosidase inhibitor.

OR and 95% CI are derived from conditional logistic regression analyses for heart failure.

^*^P-value in the multivariable model.
